# Supplementary material for: Endogenous oxytocin exerts anti-nociceptive and anti-inflammatory effects in rats
Source: Commun Biol. 2022 Sep 5;5:907. doi: 10.1038/s42003-022-03879-8 (PMC9445084; doi:10.1038/s42003-022-03879-8)
Supplement: Supplementary file 2 — Supplementary Information [file 42003_2022_3879_MOESM2_ESM.pdf]

Supplementary Figure 1

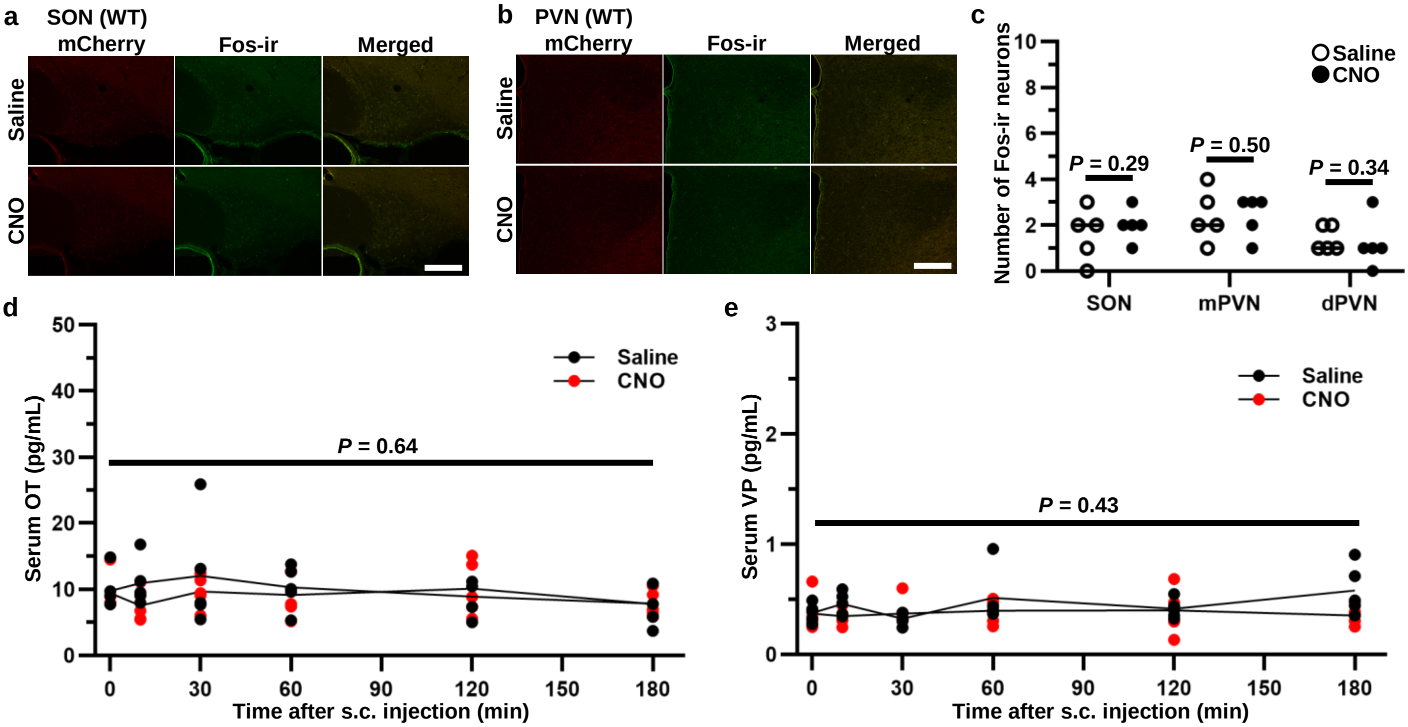

Supplementary Figure 1

S.c. injection of CNO did not affect OT neurons in the supraoptic (SON) and paraventricular nuclei (PVN) in adult male wild type (WT) rats. (Related to Fig. 2)

Fos induction was not observed at 120 min after s.c. injection of Saline and CNO (1 mg kg^{-1}) in the supraoptic (SON) **(a)** and paraventricular nuclei (PVN) **(b)** of WT rats. **(c)** Quantitative analysis of the number of Fos-ir neurons in the SON and PVN (n=10 slices from 5 rats, each). Both serum OT concentration **(d)** and VP concentration **(e)** remained unchanged after s.c. injection of Saline or CNO (1 mg kg^{-1}) in WT rats (n=5 rats in each group at each time point).

Supplementary Figure 2

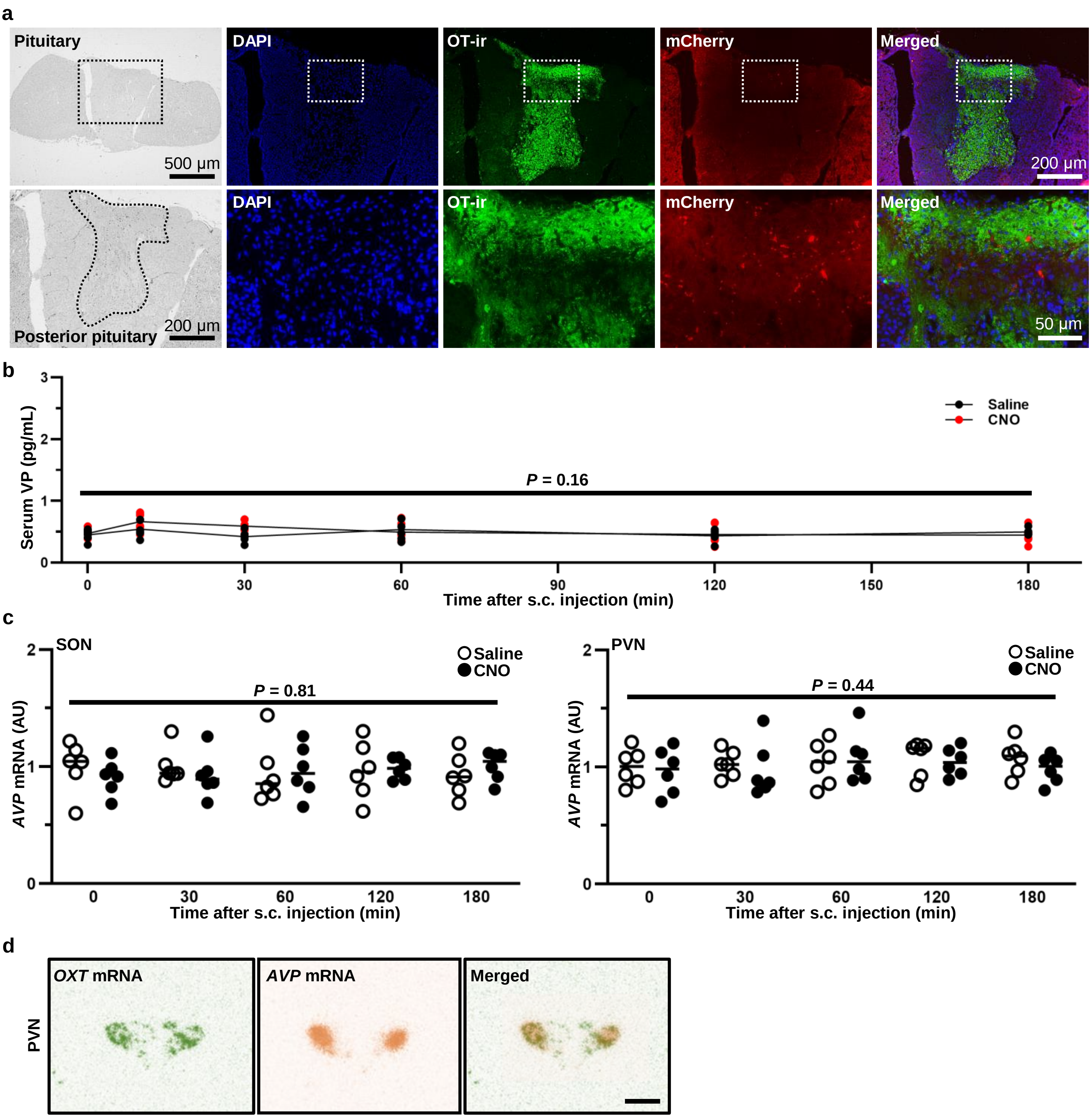

Supplementary Figure 2

hM3Dq-mCherry was observed in the posterior pituitary and serum VP concentration and gene expression of *AVP* remained unchanged after the s.c. injection of CNO in adult male OT-hM3Dq-mCherry transgenic rats. (Related to Fig. 2)

(a) Digital images of paraffin-embedded section (4 µm) of the posterior pituitary (bright field and FIHC). An mCherry fluorescence was seen in the posterior pituitary. Rectangles framed by white dotted lines are enlarged in the lower columns. **(b)** Serum VP concentration remained unchanged after the s.c. injection of CNO (1 mg kg^{-1}) in comparison to Saline (n=5 rats in each group at each time point). **(c)** Gene expression of *AVP* in the SON and PVN were comparable between Saline-injected and CNO-injected (1 mg kg^{-1}) transgenic rats (n=12 slices from 6 rats in each group at each time point). **(d)** Autoradiographed images of *in situ* hybridization (ISH) representing *OXT* (green) and *AVP* (orange) probe binding in the PVN (analyzed in two different serial sections, then images were merged). Colors were amended for distinguishing two different probes. The mRNA expression pattern was different in the PVN. These were consistent with the results of FIHC, suggesting that the ISH method used in the present study was reliable.

Supplementary Figure 3

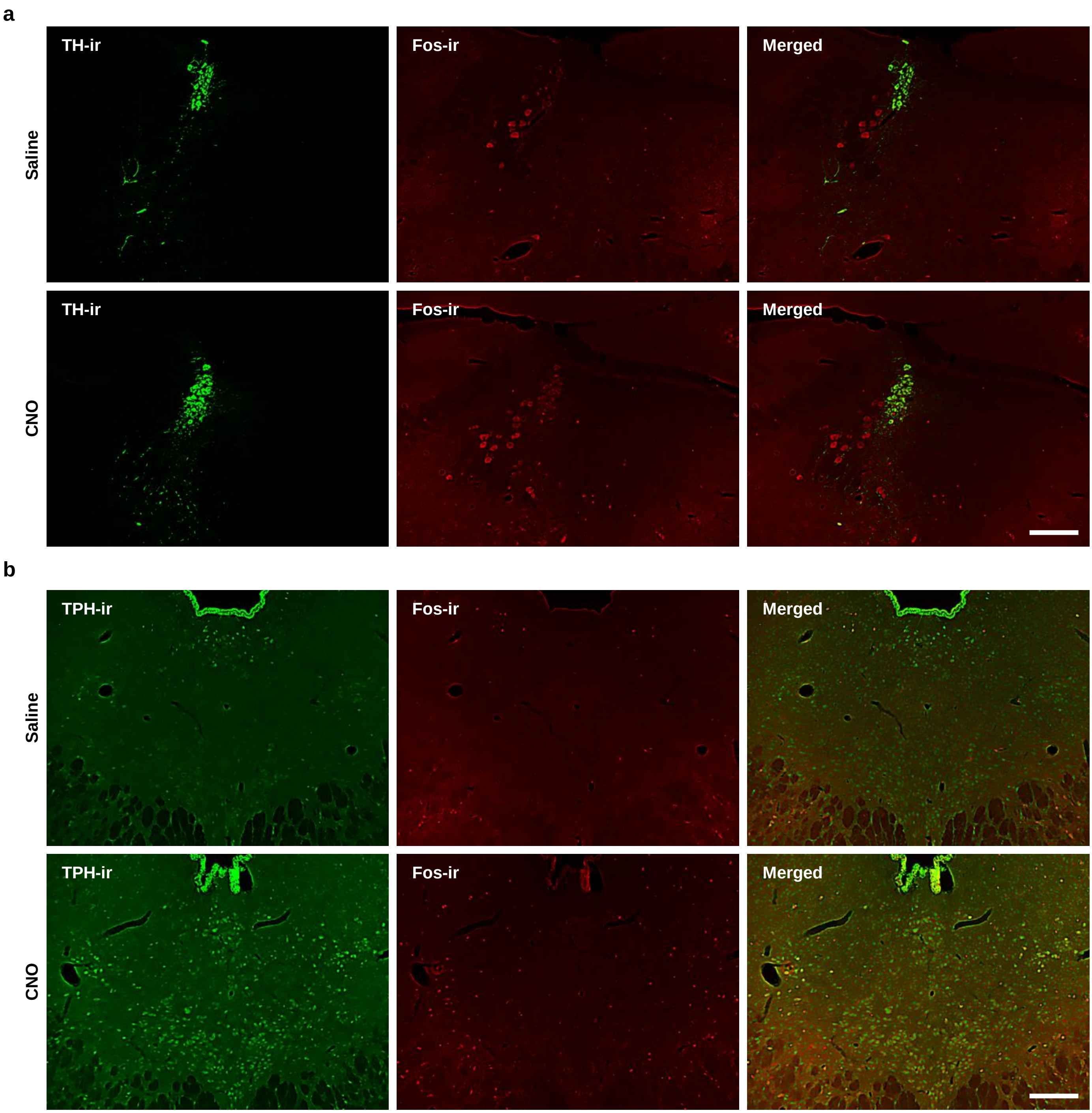

Supplementary Figure 3

FIHC images of in the locus coeruleus (LC) and dorsal raphe nucleus (DR) in adult male OT-hM3Dq-mCherry transgenic rats. (Related to Fig. 3 and 4)

Typical representative FIHC images of the locus coeruleus (LC) **(a)** and dorsal raphe nucleus (DR) **(b)** at 2 h after subcutaneous injection of Saline or CNO (1 mg kg^{-1}). Scale bars, 200 μm.

Supplementary Figure 4

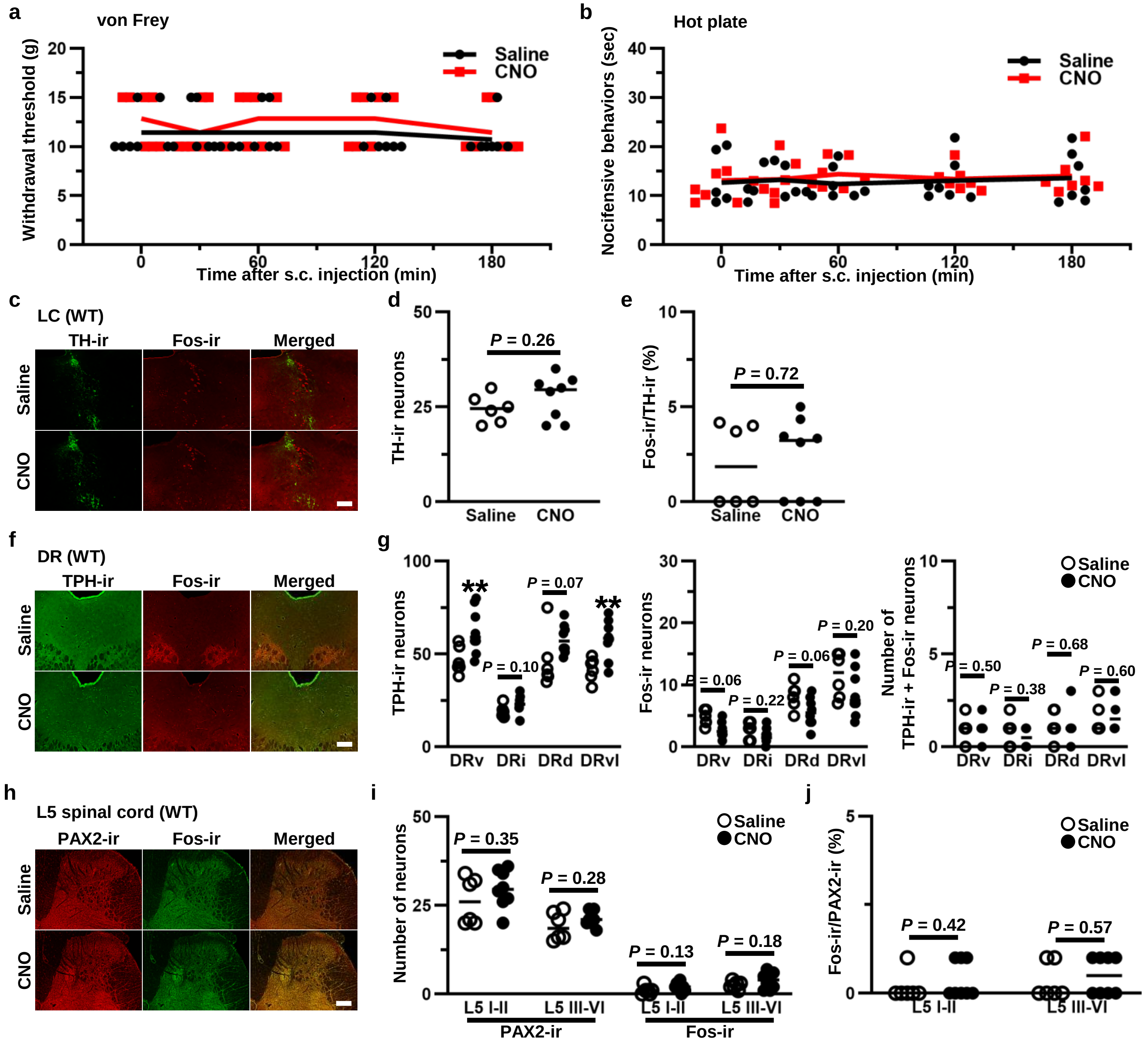

Supplementary Figure 4

Neither antinociceptive behaviours nor Fos induction in the locus coeruleus (LC), dorsal raphe nucleus (DR) and lumbar segment 5 (L5) spinal cord were altered by CNO in adult male WT rats. (Related to Fig. 3 and 4)

Mechanical/heat sensitivities evaluated by manual von Frey test **(a)** and hot plate test **(b)** were not altered after s.c. injection of CNO in WT rats, suggesting that CNO (1 mg kg^{-1}) did not affect nociceptive behaviours. Data are presented as mean ± SEM (n=5-6 rats, each in (a) and (b)). **(c)** FIHC for tyrosine hydroxylase (TH), Fos, and their merged images at 120 min after s.c. injection of Saline or CNO. **(d)** The number of TH-ir neurons was not increased after s.c. injection of CNO. **(e)** Fos induction was not observed in TH-ir neurons in the LC after s.c. injection of CNO. **(f)** FIHC for tryptophan hydroxylase (TPH), Fos, and their merged images at 120 min after s.c. injection of Saline or CNO. **(g)** The number of TPH-ir neurons was statistically increased in the DRv and DRvl, but not in the DRi and DRd. Number of Fos-ir neurons and Fos induction in TPH-ir neurons were comparable between Saline and CNO. **(h)** FIHC for PAX-2, Fos, and their merged images at 120 min after s.c. injection of Saline or CNO. **(i)** Number of PAX-2-ir neurons and Fos-ir neurons were comparable in the L5 spinal cord. **(j)** Fos induction was not observed in PAX-2-ir neurons in the L5 spinal cord after CNO injection. Scale bars, 200 μm. N=6-10 slices from 3-5 rats (c)-(j). ***P*<0.01 vs. Saline.

Supplementary Figure 5

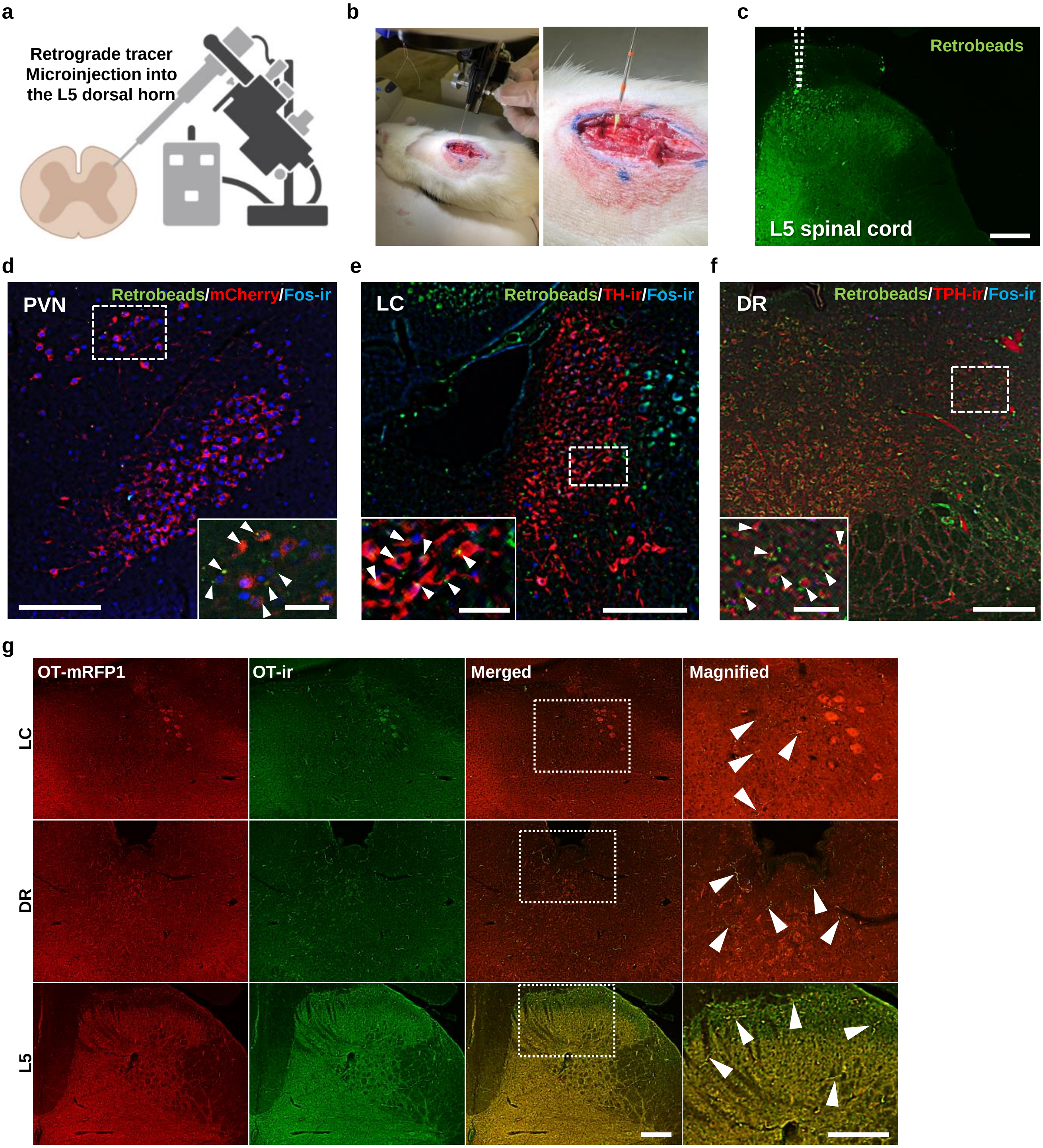

Supplementary Figure 5

Neuronal projection between the spinal dorsal horn and the dPVN, DR, and LC. (Related to Fig. 5)

(a) Schematic illustration of microinjection of retrograde tracer (Retrobeads®) into Lumbar segment 5 (L5) dorsal horn in the spinal cord. **(b)** Microinjection was implemented by dorsal approach. **(c)** Retrobeads® (green fluorescent protein, GFP) were observed in the L5 dorsal horn where the Retrobeads® were injected. GFP was observed in the dorsal paraventricular nucleus (dPVN) **(d)**, locus coeruleus (LC) **(e)**, and dorsal raphe nucleus (DR) **(f)** at 1 week after the microinjection. White arrowheads indicate GFP that was retrogressed from the L5 spinal cord. **(g)** OT-monomeric red fluorescent protein 1 (mRFP1) signal was observed in the LC, DR, and L5 spinal cord in adult male OT-mRFP1 transgenic rats. FIHC for OT was performed on the same brain/spinal cord slices to confirm that these red fluorescent signals were the axons of OT neurons. The mRFP1 signals were co-localized with OT-ir axons. Rectangles encompassed by white dotted lines are magnified in the right panels. White arrowheads indicate the axons of OT neurons. Scale bars, 200 μm and 50 μm (in magnified images).

Supplementary Figure 6

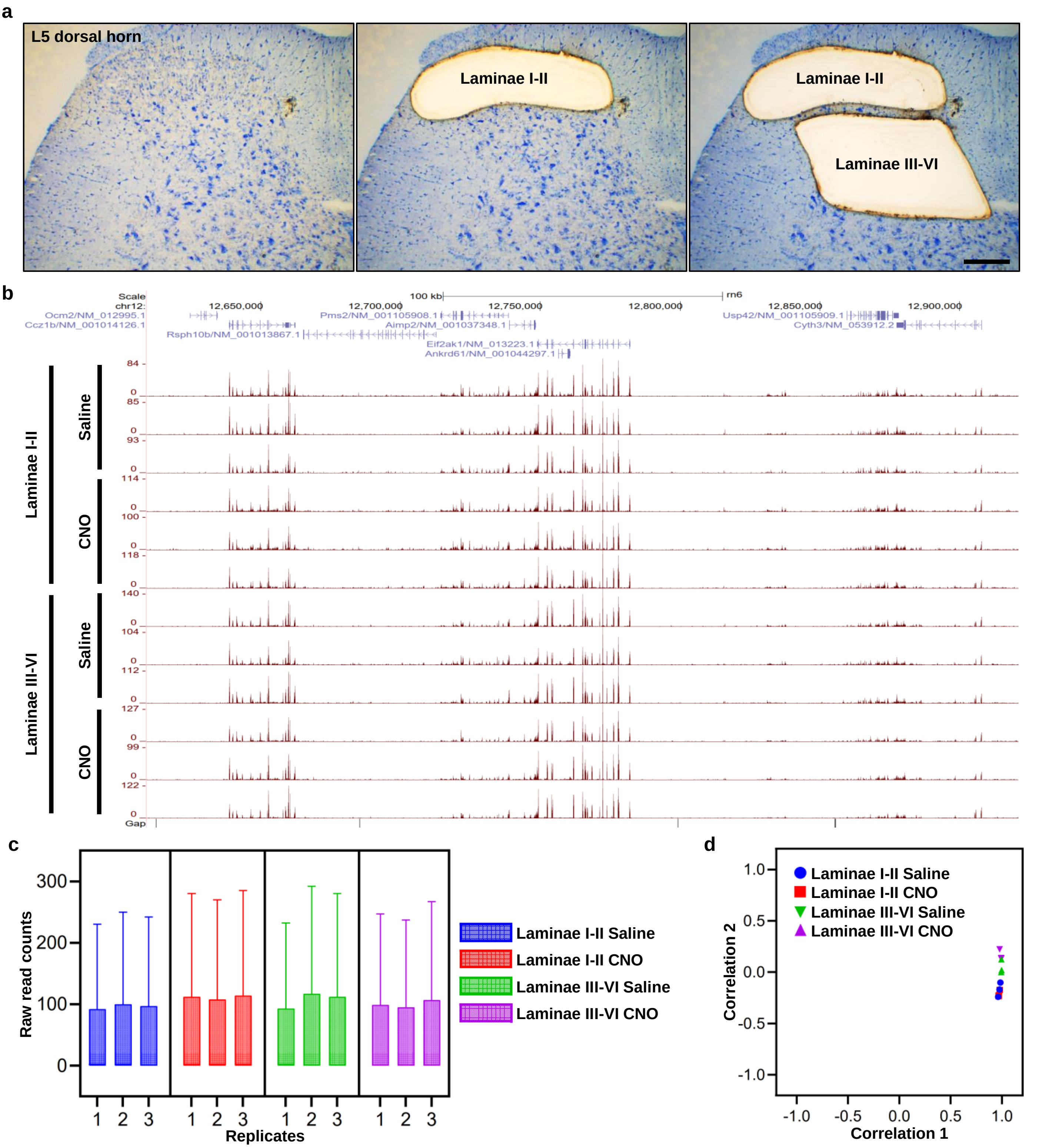

Supplementary Figure 6

Laser micro dissection (LMD) of Laminae I-II and III-VI in the L5 spinal cord for RNAseq in adult male OT-hM3Dq-mCherry transgenic rat. (Related to Fig. 5)

(a) Twelve-micrometer sections of L5 spinal cord were cut by using a cryostat. Then, the sections were stained with toluidine blue. Laminae I-II (superficial layer) and III-VI (deep layer) were anatomically determined as shown in the figure according to their morphological differences. LMD was performed using a laser microscope (Leica LMD6, Wetzlar, Germany). Scale bar, 200 μm. Read capture **(b),** read counts across all the samples box plot **(c)** and principal component analysis (PCA) plot **(d)** revealed that consistent RNAseq mapping patterns throughout the full length of known housekeeping genes, along with consistent read counts across the different samples.

Supplementary Figure 7

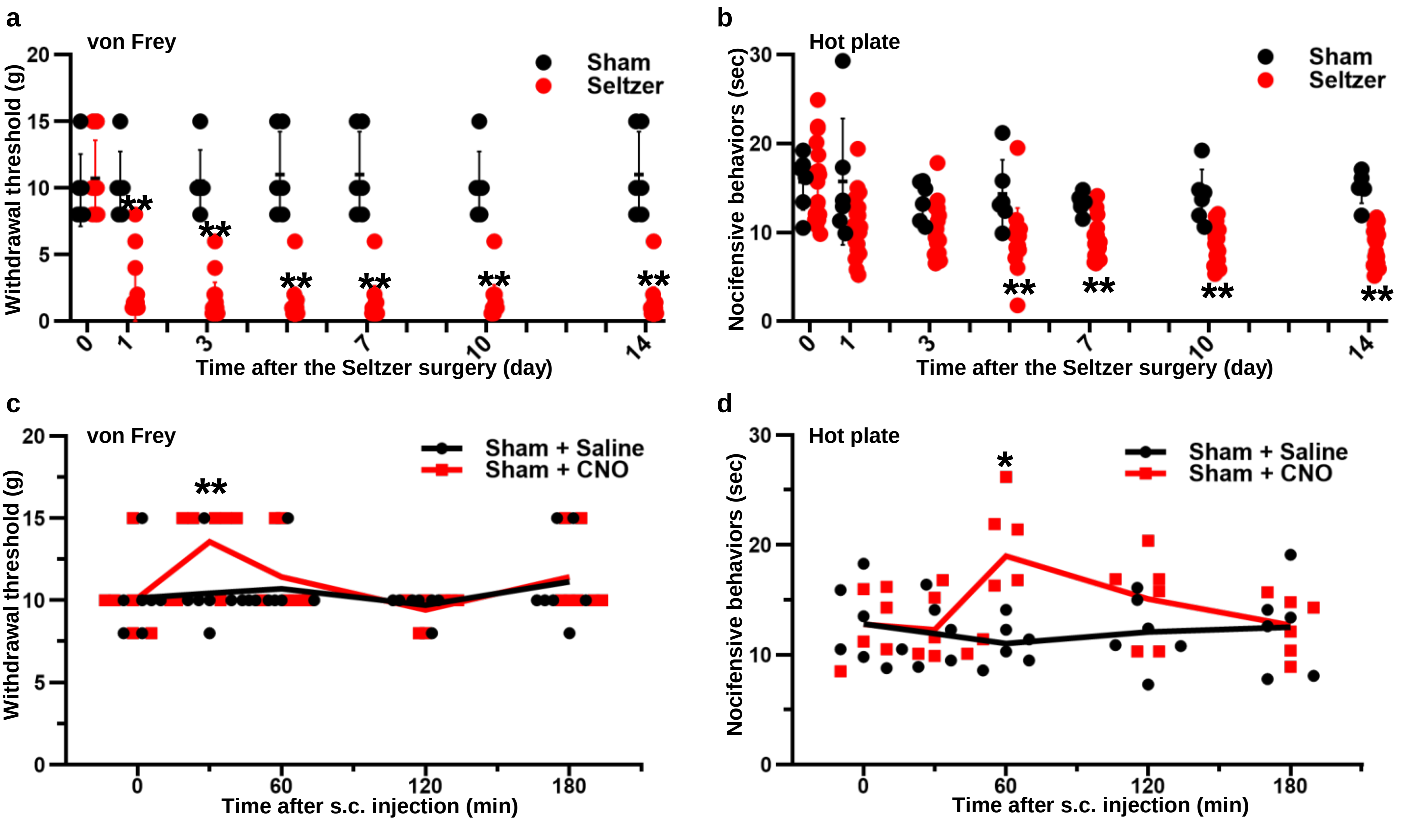

Supplementary Figure 7

Mechanical/heat hypersensitivity promptly developed in the Seltzer model in adult male OXT-hM3Dq-mCherry transgenic rats. (Related to Fig. 6)

Results of manual von Frey **(a)** and hot pate tests **(b)** after the Seltzer surgery. Based on these results, we conducted the experiment at 10 days after the Seltzer surgery. Data are presented as mean ± SEM (n=18 rats, each). ***P*<0.01 vs. Sham. Effects of Saline or CNO (1 mg kg^{-1}) on manual von Frey test **(c)** and hot pate test **(d)** after Seltzer-Sham surgery at post-operative day 10. Data are presented as mean ± SEM (n=6 rats, each). **P*<0.05, ***P*<0.01 vs. Saline at the same time point.

Supplementary Figure 8

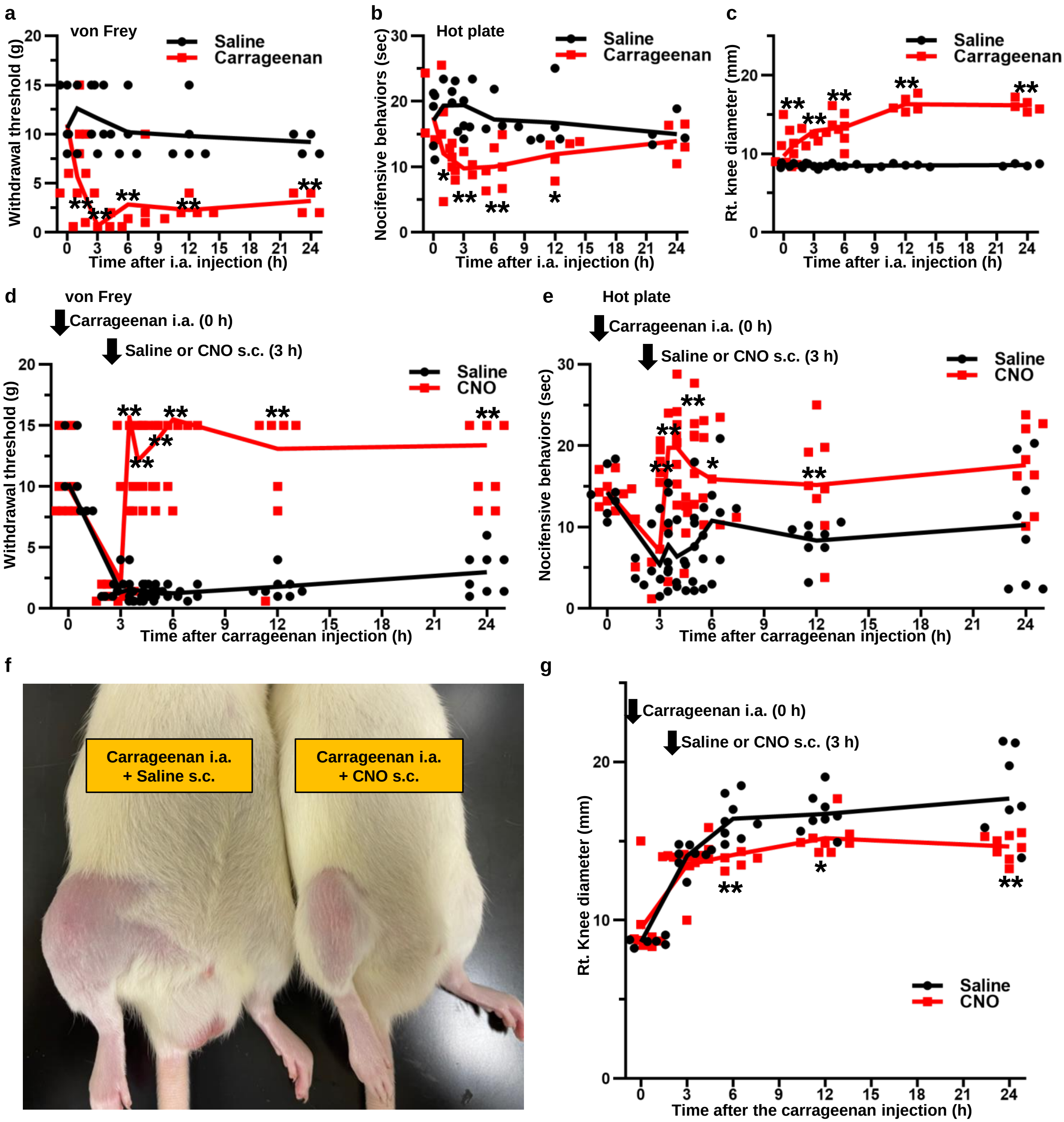

Supplementary Figure 8

Effects of CNO on von Frey test and hot plate test after intraarticular (i.a.) carrageenan treatment in adult male OXT-hM3Dq-mCherry transgenic rats. (Related to Fig. 7)

Mechanical/heat sensitivities evaluated by manual von Frey **(a)** and hot plate tests **(b)** were altered immediately after intraarticular (i.a.) injection of carrageenan (0.1 mL of 3% λ-carrageenan), reached nadir at 3 h after the injection. **(c)** Right knee diameter was significantly increased after i.a. injection of carrageenan. S.c. injection of Saline or CNO (1 mg kg^{-1}) was thus carried out at 3 h after i.a. injection. Mechanical/heat thresholds **(d, e)** were significantly elevated after CNO injection (1 mg kg^{-1}) in comparison with Saline, and prolonged effects were observed until at least at 24 h. **(f)** Digital image of right knee at 24 h after i.a. carrageenan injection, either treated with Saline or CNO. **(g)** Right knee swelling induced by carrageenan was also significantly attenuated after CNO injection. Data are presented as mean ± SEM (n=6 rats, each). *P<0.05, **P<0.01 vs. Saline.

Supplementary Figure 9

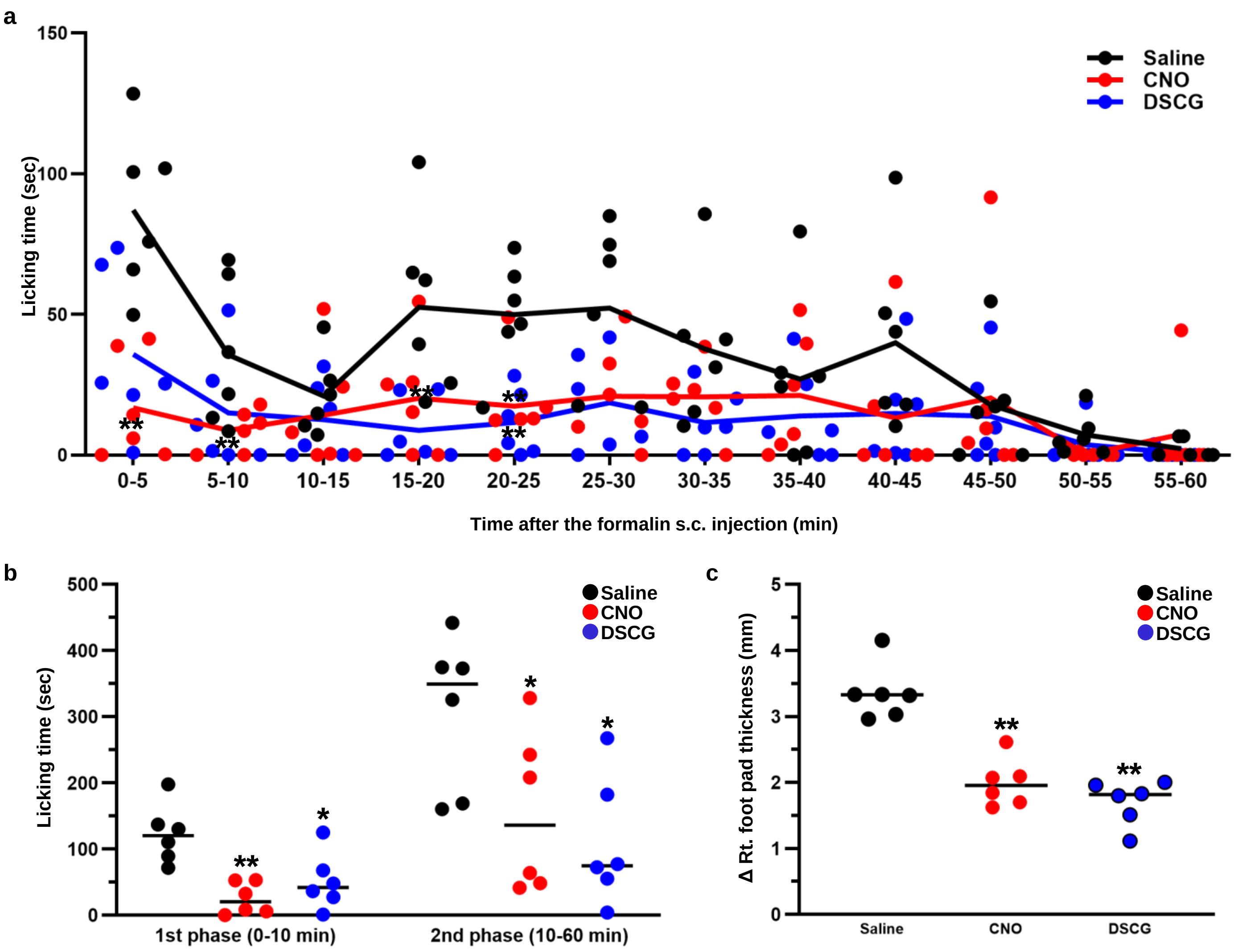

Supplementary Figure 9

Effects of CNO vs. Disodium cromoglicate (DSCG, a mast cell stabilizer) on formalin test in adult male OXT-hM3Dq-mCherry transgenic rats. (Related to Fig. 8)

Saline, CNO (1 mg kg^{-1}), or DSCG (50 mg kg^{-1}) was s.c. (systemically) injected at 30 min prior to s.c. injection of 5% formalin (100 µL) into right hind paw. Then the formalin test was performed for 60 min. Right foot pad thickness was measured at 0 and 60 min after s.c. injection of formalin. **(a)** Results of formalin test. Licking time was analyzed at 5 min interval for 60 min after s.c. injection of 5% formalin (100 µL). Data are presented as mean ± SEM (n=6 rats, each). **(b)** Total licking time was divided into 1st and 2nd phase (n=6 rats, each). **(c)** Delta right foot pad thickness compared between before and after 5% formalin (100 µL) s.c. injection (paw). *P<0.05, **P<0.01 vs. Saline.

Supplementary Figure 10

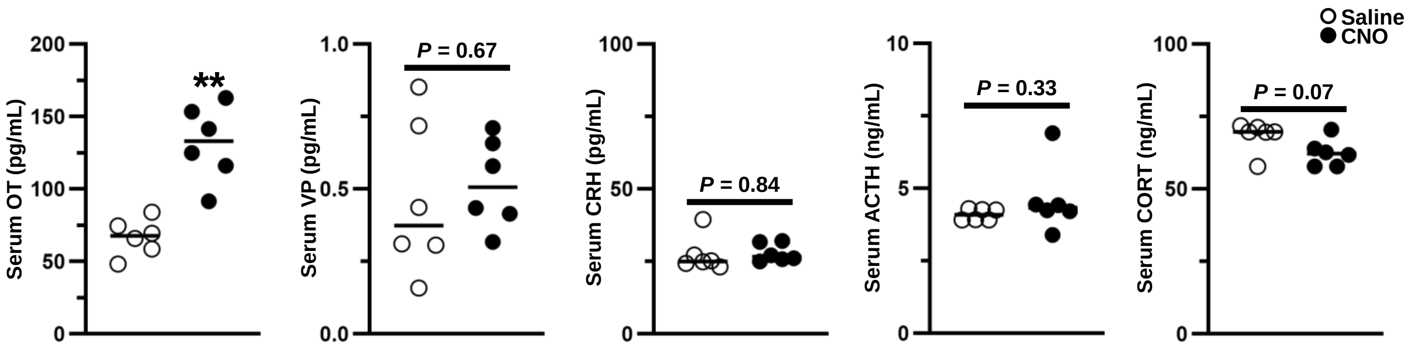

Supplementary Figure 10

Endogenous OT activation did not affect HPA axis after acute restraint stress in adult male OXT-hM3Dq-mCherry transgenic rats. (Related to Fig. 9)

Saline or CNO (1 mg kg^{-1}) was s.c. injected at 30 min prior to the experiment, then the rats were immobilized for 30 min. Just after the end of their immobilization, trunk blood samples were taken by decapitation without being anesthetized (n=6 rats, each). Serum CRH, ACTH, and CORT were analyzed by ELISA. Serum VP and OT were analyzed by RIA. See also Methods. $^{**}P<0.01$ vs. Saline.

Supplementary Table 1

Primary antibodies used in the fluorescent immunohistochemistry, Related to Methods.

Abbreviations; OT, oxytocin: VP, vasopressin: TH, tyrosine hydroxylase: TPH, tryptophan hydroxylase: CRH, corticotrophin releasing hormone.

Product (Cat. No.)	Host	Dilution	Company
**Fos (SC-52)**	rabbit	1:1000	Santa Cruz Biotechnology, Dallas, TX, USA
**Fos (SC-52G)**	goat	1:250	Santa Cruz Biotechnology, Dallas, TX, USA
**OT (AB911)**	rabbit	1:10,000	Chemicon, Temecula, CA, USA
**VP (403 004)**	guinea pig	1:500	Synaptic System, Göttingen, Germany
**TH (GTX113016)**	rabbit	1:200	GeneTex, Irvine, CA, USA
**TPH (bs-5601R)**	rabbit	1:100	Bioss Antibodies, Woburn, MA, USA
**PAX2 (H00005076-M01)**	mouse	1:500	Abnova, Taipei, Taiwan
**CRH (T-4037)**	rabbit	1:100	BMA BIOMEDICALS, Augst, Switzerland

Supplementary Table 2
Secondary antibodies used in the fluorescent immunohistochemistry, Related to Methods.

Product (Cat. No.)	Host	Dilution	Immunogen	Company
**Alexa Fluor 405 (ab175651)**	donkey	1:500	anti-rabbit	abcam, Cambridge, UK
**Alexa Fluor 488 (A-21206)**	donkey	1:500	anti-rabbit	Thermo Fisher Scientific, Waltham, MA, USA
**Alexa Fluor 488 (A-11008)**	goat	1:500	anti-rabbit	Thermo Fisher Scientific, Waltham, MA, USA
**Alexa Fluor 568 (A-10037)**	donkey	1:500	anti-mouse	Thermo Fisher Scientific, Waltham, MA, USA
**Alexa Fluor 568 (A-11057)**	donkey	1:500	anti-goat	Thermo Fisher Scientific, Waltham, MA, USA
**Alexa Fluor 568 (A-11011)**	goat	1:500	anti-rabbit	Thermo Fisher Scientific, Waltham, MA, USA
**Alexa Fluor 633 (A-21105)**	goat	1:500	anti-gunia pig	Thermo Fisher Scientific, Waltham, MA, USA

Supplementary Table 3

Oligoprobe sequences used in the *in situ* hybridization histochemistry, Related to Methods.

Abbreviations; OXT, oxytocin: AVP, vasopressin: TH, tyrosine hydroxylase: TPH2, tryptophan hydroxylase 2: CRH, corticotrophin releasing hormone: POMC, pro-opiomelanocortin.

Probe	Antisense sequence
***OXT* mRNA**	5'- CTC GGA GAA GGC AGA CTC AGG GTC GCA GGC -3′
***AVP* mRNA**	5'- CAG CTC CCG GGC TGG CCC GTC CAG CT -3′
***TH* mRNA**	5'- GAC AAG CTC AGG AAC TAT GCC TCT CGT ATC -3'
***TPH2* mRNA**	5’- TCC GTC CAA ATG TTG TCA GGT GGA TTC AGC GTC ACA ATG GTG GTC -3’
***CRH* mRNA**	5'- CAG TTT CCT GTT GCT GTG AGC TTG CTG AGC TAA CTG CTC TGC CCT GGC -3′
***POMC* mRNA**	5'- TGG CTG CTC TCC AGG CAC CAGCTC CAC ACA TCT ATG GAG G -3'
